# Loss of heterozygosity at 9q33 and hypermethylation of the *DBCCR1* gene in oral squamous cell carcinoma

**DOI:** 10.1038/sj.bjc.6601980

**Published:** 2004-06-29

**Authors:** S Gao, J Worm, P Guldberg, H Eiberg, A Krogdahl, J A Sørensen, C-J Liu, J Reibel, E Dabelsteen

**Affiliations:** 1 Department of Oral Diagnostics, School of Dentistry, University of Copenhagen, Nørre Alle 20, DK-2200 Copenhagen, Denmark; 2 Institute of Cancer Biology, Danish Cancer Society, DK-2100 Copenhagen, Denmark; 3 Institute of Medical Biochemistry and Genetics, University of Copenhagen, DK-2200 Copenhagen, Denmark; 4 Department of Pathology, Odense University Hospital, DK-5000 Odense, Denmark; 5 Department of Plastic Surgery, Odense University Hospital, DK-5000 Odense, Denmark; 6 Department of Dentistry, Mackay Memorial Hospital, Taipei

**Keywords:** *DBCCR1*, oral carcinoma, DNA methylation, loss of heterozygosity, chromosome 9q

## Abstract

The *DBCCR1* gene at chromosome 9q33 has been identified as a candidate tumour suppressor, which is frequently targeted by promoter hypermethylation in bladder cancer. Here, we studied the possible involvement of *DBCCR1* in the development of oral squamous cell carcinoma. DNA from 34 tumours was examined for loss of heterozygosity (LOH) at three markers surrounding *DBCCR1* and for hypermethylation of the *DBCCR1* promoter, using methylation-specific PCR and methylation-specific melting-curve analysis. LOH was found in 10 of 31 cases (32%), and *DBCCR1* hypermethylation was present in 15 of 34 cases (44%). Hypermethylation of *DBCCR1* was also present in three of seven epithelial tissues adjacent to the tumours, including two hyperplastic and one histologically normal epithelia. Furthermore, of four oral leukoplakias with dysplasia, one showed LOH at 9q33 and two showed *DBCCR1* hypermethylation. These data suggest that LOH at 9q33 and hypermethylation of the *DBCCR1* promoter are frequent and possibly early events in oral malignant development.

Oral cancer comprises about 3% of all newly diagnosed cancer cases in the Western countries. Despite advances in therapy, the 5-year survival rate after diagnosis is still poor and remains ∼50% (Landis *et al*, 1999; Silverman, 2001). Clinically, oral carcinomas often develop in a two-step process. The first step is characterised by the appearance of potentially malignant lesions such as leukoplakias and erythroplakias, and the second step is characterised by the development of carcinomas. However, clinical and histopathological features are insufficient measures for predicting the prognosis of potentially malignant lesions (Warnakulasuriya, 2000, 2001). Furthermore, a recent study indicated that clinically and histologically normal mucosa adjacent to tumours may harbour patches of genetically altered cells (Braakhuis *et al*, 2003). It is, therefore, important to find molecular markers for identifying the minor fraction of oral lesions that will develop into carcinoma.

Loss of heterozygosity (LOH) at multiple chromosome regions and genetic and epigenetic alterations of several proto-oncogenes and tumour suppressor genes have been demonstrated in oral carcinomas, including alterations of the *TP53*, *p16*, *p15*, *MGMT, E-cadherin* genes and *RAS* (Califano *et al*, 1996; Partridge *et al*, 1999; Williams, 2000; Ogi *et al*, 2002; Viswanathan *et al*, 2003). In addition, our previous study showed that hypermethylation of the *ABO* gene promoter was associated with loss of expression of A/B antigen in approximately one-third of oral squamous cell carcinomas (Gao *et al*, 2004). LOH at 9q34, in which the *ABO* gene is located, was also a frequent event in these tumours. However, a number of tumours from AO and BO heterozygotes showed deletion of the O allele, which does not encode a functional glycosyltransferase, suggesting the existence of an additional tumour suppressor gene on chromosome 9q. The *DBCCR1 (deleted in bladder cancer chromosomal region candidate 1)* gene at chromosome 9q33 was identified as a putative tumour suppressor gene that is frequently targeted by hypermethylation in transitional cell carcinomas of the bladder (Habuchi *et al*, 1997, 1998, 2001; Nishiyama *et al*, 1999). There are, at present, no reports of *DBCCR1* alterations in other cancers. The aim of this study was to examine for LOH at the 9q33 region and determine the methylation status of *DBCCR1* in oral squamous cell carcinomas and potentially malignant oral lesions.

## MATERIALS AND METHODS

### Sample preparation

Surgical specimens of oral lesions were obtained from School of Dentistry, National Yang-Ming University, Taipei, and Odense University Hospital, Denmark. The median age of the patients was 60 years (range 35–89 years); there were six women and 32 men. The materials included unfixed frozen tissues from 34 patients with oral squamous cell carcinoma and four patients with potentially malignant lesions (leukoplakia with epithelial dysplasia). A laser microdissection system (PALM) was used to separate tumour cells or leukoplakia epithelium from normal connective tissue. In seven cases, tumour-adjacent epithelium was isolated as well. DNA was extracted by routine procedures using the DNeasy Kit (Qiagen). Informed consent and approval by the Ethics Committee were obtained according to Danish legislation.

### LOH analysis

DNA from tumour or leukoplakia lesions and matched normal tissues was screened for LOH at the 9q33 region using the three microsatellite markers, D9S195, D9S1872 (http://www.gdb.org) and 9-11407. The latter marker was designed by one of us (HE) and is located ∼300 kb upstream of exon 1 of the *DBCCR1* gene, according to GenBank accession no. AY438564. The primer sequences of 9-11407 were 5′-CAACAAAGTCAATCCCAGCA-3′ and 5′-GGTTCACTAAGAGCACAATTGTTTA-3′. PCR was performed using a ^33^P end-labelled primer, and the amplified fragments were separated by electrophoresis in a 6% denaturing polyacrylamide gel, as described elsewhere (Gao *et al*, 2004). LOH was determined as at least a 50% reduction in the relative intensity of one allele compared with the normal control. Control samples were included during all procedures.

### Methylation analysis

Genomic DNA was treated with sodium bisulphite as described previously (Clark *et al*, 1994). For methylation-specific PCR (MS-PCR) analysis of the *DBCCR1* promoter (GenBank accession no. AF027734), the primers for the unmethylated reaction were 5′-TTTATGGTTGTAAATTGATTTGGTGTGT-3′ and 5′-CAACTCACATTCCAAACACAACACA-3′, which amplify a 269-bp product (positions 15–283), and the primers for the methylated reaction were 5′-TTGTAAATTGATTTGGCGCGC-3′ and 5′-TTCCGAACACGACGCGAAA-3′, which amplify a 253-bp product (positions 22–274). PCR was carried out using the HotStarTaq Kit (Qiagen); the annealing temperatures for the unmethylated and methylated reactions were 60 and 62°C, respectively. Primer sequences and reaction conditions for MS-PCR analysis of the *ABO* gene promoter were as described (Kominato *et al*, 1999; Gao *et al*, 2004). The PCR products were resolved on 2% agarose gels. DNA treated with *Sss*I methyltransferase (New England Biolabs) served as the methylated control.

For methylation-specific melting-curve analysis (MS-MCA) of *DBCCR1*, the primers were 5′-GGGAGGTAGAGGGAGTAGTGAT-3′ and 5′-AAAATACCTAACTCCTAACAACCTAAC-3′, which amplify a 117-bp product (positions 127–243). PCR and subsequent MCA were carried out as previously described (Worm *et al*, 2001) using the LightCycler (Roche) and the FastStart DNA Master SYBR Green I Kit (Roche). Reactions were started by initial denaturation at 95°C for 10 min, followed by cycling at 95°C for 10 s, a transition from 72 to 66°C at 0.5°C cycle^−1^ for 10 s and 72°C for 20 s. Melting-curve analysis was performed immediately after amplification by measuring the fluorescence of SYBR Green I during a linear temperature transition from 70 to 95°C at 0.05°C s^−1^. Fluorescence data were converted into melting peaks by the LightCycler software (Ver.3.39) by plotting the negative derivative of fluorescence over temperature *vs* temperature (−d*F/*d*T vs T*).

### Statistical analysis

Correlation analyses were performed using Fisher's exact probability test.

## RESULTS

### LOH analysis of chromosome 9q33

LOH analysis of the 9q33 region using three microsatellite markers showed allelic loss in 10 of 31 (32%) informative cases of oral squamous cell carcinoma (Table 1

**Table 1:** Hypermethylation of *DBCCR1* and LOH at 9q33 in oral squamous cell carcinomas and leukoplakias with dysplasia

				**Hypermethylation**		**LOH**		
	**Case #**	**Sex**	**Age**	***DBCCR1***	***ABO***	**D9S1872**	**D9S195**	**9-11407**
	Carcinomas							
1	CT5	M	54	+	−	−	−	+
2	CT6	M	56	−	−	−	−	−
3	CT7	M	60	+	−	−	−	−
4	CT8	M	62	−	−	+	−	−
5	CT10	M	57	−	+	NA	NA	NA
6	CT11	M	57	−	+	−	−	−
7	CT12	M	50	−	−	−	−	−
8	CT14	M	50	+	−	−	−	−
9	CT15	M	53	−	−	−	−	−
10	CT16	M	37	+	−	−	−	−
11	CT17	M	57	−	−	−	−	−
12	CT18	M	65	+	+	−	+	−
13	CT19	M	58	−	−	−	−	−
14	CT20	M	54	−	−	−	−	−
15	CT21	M	71	−	−	−	−	−
16	CT22	M	65	−	−	−	−	−
17	CT23	M	35	+*	+*	−	−	−
18	CTGx	F	57	+	+	+	+	+
19	30365	M	66	+	−	NA	NA	NA
20	19395	M	61	+	−	−	−	+
21	25941	M	65	−	−	−	−	−
22	15374	M	69	−	+	−	−	−
23	18034	M	76	−	−	NA	NA	NA
24	31572T1	F	55	+	+	−	+	−
	31572T2			+	+	+	+	+
25	33379	M	84	−	−	−	−	−
26	28753	F	52	+	−	−	+	+
27	19274	M	61	−	−	−	+	−
28	2132	M	58	+*	+	−	−	−
29	1592	F	71	+	−	−	−	−
30	17093	M	60	−	−	+	+	+
31	29627	M	61	−	−	−	−	−
32	21394	M	69	+	+	−	−	−
33	27088	F	89	+*	+*	−	−	+
34	33103	M	69	−	+	−	−	−
	Leukoplakias with dysplasia							
35	6042	F	60	−	−	−	−	−
36	24710	M	54	−	−	+	−	−
37	24722	M	53	+	−	−	−	−
38	16050	M	51	+	−	−	−	−

NA=no available information; T1=well-differentiated tumour cells adjacent to normal epithelium.

T2=poor-differentiated tumour cells far away from normal epithelium.

* Hypermethylation was found in both normal epithelia and tumour cells.

; see Figure 1

**Figure 1:**
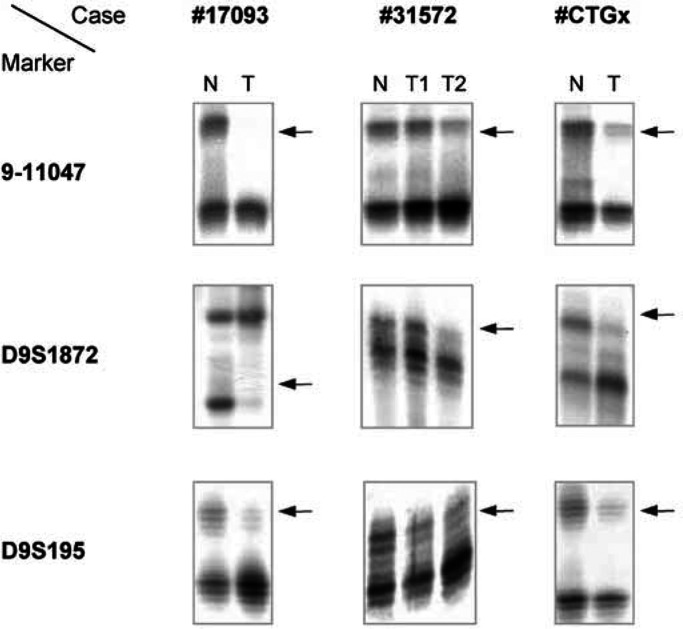
LOH analysis of 9q33 in oral squamous cell carcinomas. T, tumour; N, normal tissue; T1, well-differentied tumour cells adjacent to normal epithelium; T2, poor-differentied tumour cells far away from normal epithelium. Arrows indicate LOH.

for examples). Among these, four showed LOH at D9S1872, six at D9S195, and seven at 9-11407. Notably, three cases showed LOH at 9-11407 located ∼300 kb upstream of *DBCCR1*, but retention of D9S195 located in intron 1 of *DBCCR1*. In one case, in which DNA was isolated from both well- and poor-differentiated tumour cells from the same tumour, LOH at D9S195 was found in both populations, but only the poor-differentiated tumour cells showed LOH at 9-11407 and D9S1872 (Figure 1). LOH at D9S1872 was also found in one of four leukoplakias with dysplasia. No LOH was found in epithelial tissues adjacent to the tumours.

### Methylation analysis

**Figure 2:**
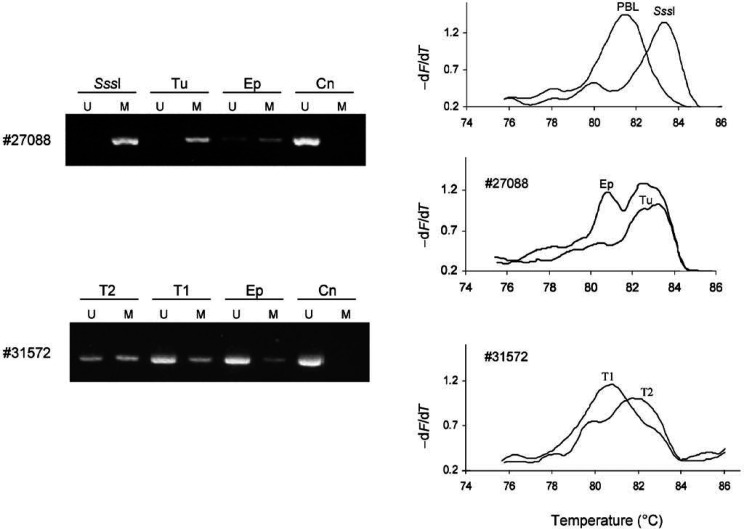
Methylation analysis of the *DBCCR1* gene promoter in oral squamous cell carcinomas. Left, MS-PCR. Genomic DNA was treated with sodium bisulphfite and PCR-amplified with primer pairs specific for methylated (M) and unmethylated (U) alleles. Right, MS-MCA. Bisulphfite-treated DNA was amplified in the presence of SYBR Green I using primers that do not discriminate between methylated and unmethylated *DBCCR1* alleles. The melting characteristics of the PCR products were determined directly in the PCR tube by continuous fluorescence monitoring during a temperature transition. *Sss*I-methylated DNA and genomic DNA from normal peripheral blood lymphocytes (PBL) provided positive controls for methylated and unmethylated *DBCCR1* alleles, respectively. Tu, tumour; Ep, epithelium; Cn, connective tissue; T1, well-differentiated tumour cells adjacent to normal epithelium; T2, poor-differentiated tumour cells far away from normal epithelium.

Hypermethylation of the *DBCCR1* gene promoter was present in 15 out of 34 (44%) oral squamous cell carcinomas, as determined by MS-PCR analysis (Table 1; see Figure 2

for examples). In three out of seven cases, *DBCCR1* hypermethylation was also found in tumour-adjacent tissues, including two hyperplastic and one histologically normal epithelia. To further characterise *DBCCR1* methylation patterns in oral carcinomas and to exclude possible false-positive MS-PCR results, all samples showing a positive signal for methylated *DBCCR1* alleles using MS-PCR were also examined using MS-MCA (Figure 2). Aberrant methylation was confirmed in all cases. However, in one case (#31572), well- and poor-differentiated tumour cells isolated from the same lesion showed different methylation patterns (Figure 2). Hypermethylation of the *DBCCR1* gene was also found in two of four leukoplakias with dysplasia, none of which showed LOH at 9q33 (Table 1).

Concomitant LOH at 9q33 and hypermethylation of the *DBCCR1* gene were found in seven carcinomas (*P*=0.057); however, this correlation was only significant for microsatellite marker 9-11407, which is located ∼300 kb upstream of exon 1 of the *DBCCR1* gene (Table 2

**Table 2:** Correlation analysis of LOH at 9q33 and *DBCCR1* and *ABO* hypermethylation

			**Methylation of *DBCCR1***		
		**No**	**M (%)**	**U (%)**	***P*-value**
Methylation of *ABO*	M	11	7 (63.6)	4 (36.4)	
	U	23	8 (34.8)	15 (65.2)	0.11
LOH at 9q33	+	10	7 (70.0)	3 (30.0)	
	−	24	8 (33.3)	16 (67.7)	0.057
at 9-11047	+	7	6 (85.7)	1 (14.3)	
	−	27	9 (33.3)	18 (67.7)	0.018
at D9S1872	+	4	2 (50.0)	2 (50.0)	
	−	30	13 (43.3)	17 (56.7)	0.60
at D9S195	+	6	4 (66.7)	2 (33.3)	
	−	28	11 (39.3)	17 (61.7)	0.22

M=hypermethylation; U=no methylation.

). Hypermethylation of *ABO* was found in 11 out of 34 (32%) tumour samples and in three adjacent epithelia (Table 1) (Gao *et al*, 2004), but there was no correlation between the *DBCCR1* and *ABO* hypermethylation events (P = 0.11; Tables 1 and 2).

## DISCUSSION

Substantial evidence suggests that aberrant hypermethylation of promoter CpG islands may constitute an alternative mechanism to intragenic mutations and deletions for inactivation of tumour suppressor genes (Worm and Guldberg, 2002; Nephew and Huang, 2003). Hypermethylation of the *DBCCR1* gene as well as LOH and homozygous deletions at the *DBCCR1* locus have been shown to be frequent events in bladder cancer (Fujiwara *et al*, 2001; Habuchi *et al*, 1998, 2001; Nishiyama *et al*, 1999). Previous studies of head and neck carcinomas have demonstrated LOH involving the 9q32-33 region, which covers the *DBCCR1* gene (Ah-See *et al*, 1994). In the present study, LOH at 9q33 was found in 32% of oral squamous cell carcinomas, suggesting that this region contains a tumour suppressor gene involved in oral carcinogenesis. Notably, LOH frequently involved microsatellite marker D9S195, which is located in intron 1 of *DBCCR1* and was originally used to identify this gene as a candidate tumour suppressor (Habuchi *et al*, 1997). Methylation analysis of the *DBCCR1* promoter region using two different techniques showed aberrant hypermethylation in 44% of the tumours. These data add *DBCCR1* to the list of tumour suppressor genes known to be targeted by promoter hypermethylation in oral carcinomas, including *p16*, *p15*, *E-cadherin*, *MGMT* and *ABO* (Akanuma *et al*, 1999; Kim *et al*, 2000; Yakushiji *et al*, 2001; Chang *et al*, 2002; Hasegawa *et al*, 2002; Viswanathan *et al*, 2003; Gao *et al*, 2004). No correlation was found between hypermethylation of *DBCCR1* at 9q33 and *ABO* at 9q34, suggesting that these genes are epigenetically targeted in oral carcinogenesis by independent and possibly specific events.

Genetic and epigenetic alterations of the *DBCCR1* gene were not restricted to oral carcinomas. LOH at 9q33 was also demonstrated in one of four patients with severe epithelial dysplasia, and *DBCCR1* hypermethylation was present in another two of these four cases. Aberrant hypermethylation levels were found even in tumour-adjacent epithelia with no histopathological evidence of malignancy, suggesting that it may represent an early event in oral malignant development. In bladder cancer, field cancerisation has been attributed to age-related methylation of *DBCCR1* in normal epithelium (Habuchi *et al*, 2001). The presence of *DBCCR1* hypermethylation in oral tumour-adjacent epithelium is of great interest and should be further investigated in order to elucidate whether local recurrence or field cancerisation in oral cancer patients can be explained, at least in some cases, by the existence of a *DBCCR1*-hypermethylated field in histologically normal epithelium.

In the present work, we were not able to detect any divergence between the two groups of patients, which were of different ethic origin and exposed to different environmental factors (betel/tobacco and alcohol/tobacco). However, the material is too limited to make any firm conclusions. In a new prospective study, we are investigating whether the methylation and LOH status have a clinical significance.

There is still little information about the possible function of the *DBCCR1* gene in carcinogenesis. Unresolved issues include the apparent lack of *DBCCR1* expression in most normal tissues and the unclear correlation between hypermethylation and transcriptional silencing of this gene (Habuchi *et al*, 1998), questioning the role of *DBCCR1* as a tumour suppressor in the homeostasis of normal cells. Previous cell cycle studies suggested that *DBCCR1* has growth-suppressing and antiproliferative activities mediated via modulation of the G_1_ checkpoint. Overexpression of *DBCCR1* caused a slower G_1_ transition rather than G_1_ arrest and did not affect apoptosis (Nishiyama *et al*, 2001). Although these functional studies and the high rate of *DBCCR1* hypermethylation in oral squamous cell carcinomas support the candidacy of *DBCCR1* as a tumour suppressor at 9q33, additional studies are required to unravel its possible role in oral malignant development.
